# Supplementary material for: Emergence of the Ug99 lineage of the wheat stem rust pathogen through somatic hybridisation
Source: Nat Commun. 2019 Nov 7;10:5068. doi: 10.1038/s41467-019-12927-7 (PMC6838127; doi:10.1038/s41467-019-12927-7)
Supplement: Supplementary file 1 — Supplementary Information [file 41467_2019_12927_MOESM1_ESM.pdf]

Supplementary Information

Emergence of the Ug99 lineage of the wheat stem rust pathogen through somatic hybridisation

Li *et al.*

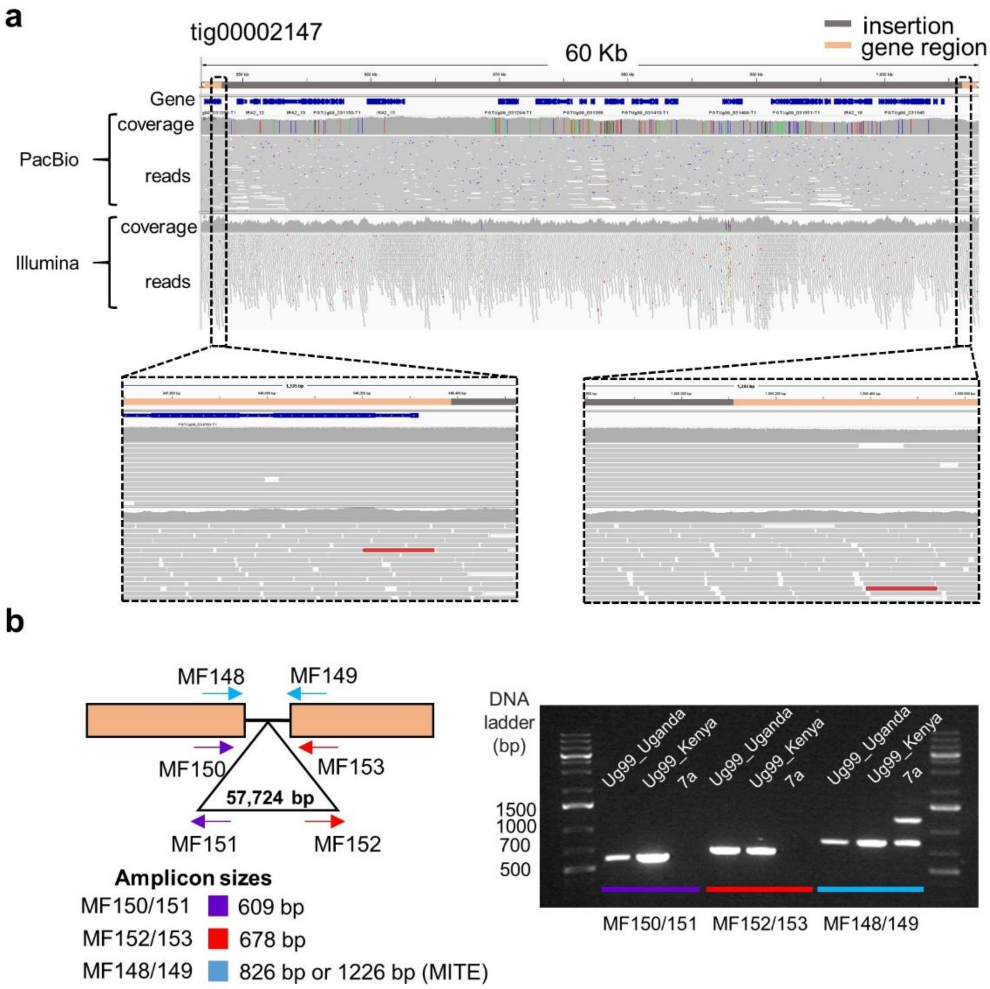

Supplementary Fig. 1 | Presence of a 57 kbp-insert in one allele of *AvrSr35* in Ug99. a, Genome browser view of a 60 kbp genomic region in haplotype C of Ug99. The top bar shows the *AvrSr35* coding sequences (orange) flanking a 57 kbp-insert (grey). Annotated gene models (blue) are shown below. The following tracks show the read coverage graph and the alignments of Ug99 reads mapped to this region. Zoomed-in areas (boxed) show read mapping across the junction between the *AvrSr35* coding sequence and the 5’ and 3’ ends of the inserted sequence. **b,** Validation of 57 kbp-insert in *AvrSr35* of Ug99 isolates via PCR amplification. The positions of primers on the *AvrSr35* gene (orange boxes) and insertion (triangle) are shown along with the predicted amplicon sizes. PCR amplification products from the original Ug99 isolate (Ug99_Uganda), the Kenyan Ug99 isolate 04KEN156/04 (Ug99_Kenya)[1,2] and the isolate CRL 75-36-700 (7a)[3]. Note that 7a is heterozygous for a wildtype allele of *AvrSr35* and a virulence allele containing a 400bp MITE insertion[2].

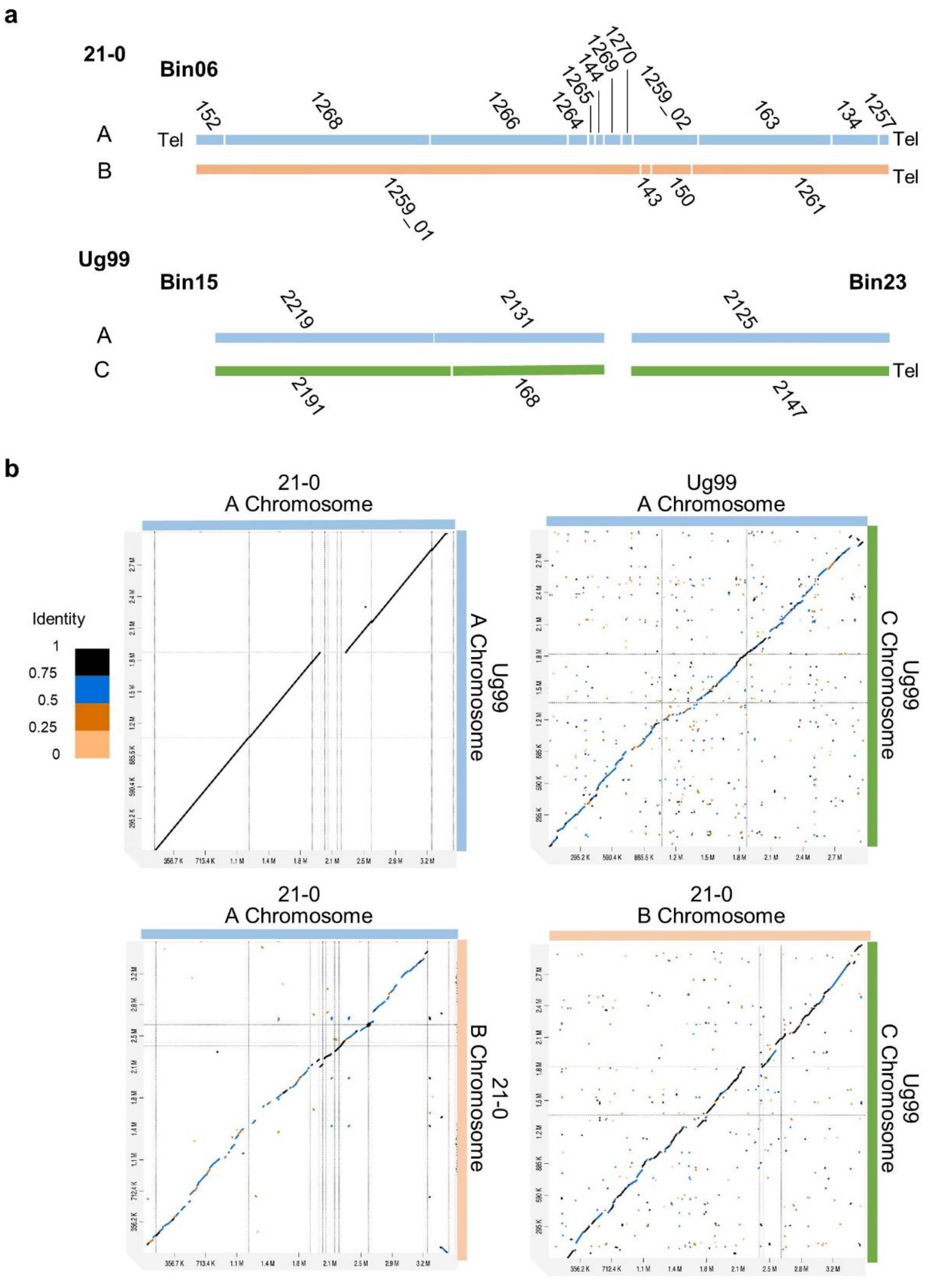

Supplementary Fig. 2 | One of the homologous chromosomes containing *AvrSr50* and *AvrSr35* loci is nearly identical in *Pgt*21-0 and Ug99. a, Schematic representation of the alignment of contigs in *Pgt*21-0 and Ug99 derived from the homologous chromosomes. Contig IDs are indicated as numbers and presence of telomeres as “Tel”. The *Pgt*21-0 contigs were assembled as Bin06 and contain telomeres at both ends indicating that a full chromosome was represented. The homologous contigs from Ug99 were present in two bins (Bin15 and Bin23). Contigs are coloured according to haplotype designation; A (light blue); B (orange); C (green). **b,** Dot plots of alignments between the homologous chromosomes of each haplotype, indicated by coloured bars at the top and right. X- and y-axes represent nucleotide positions. Colour key indicates sequence identity fraction for all dot plots (maximum identity score =1).

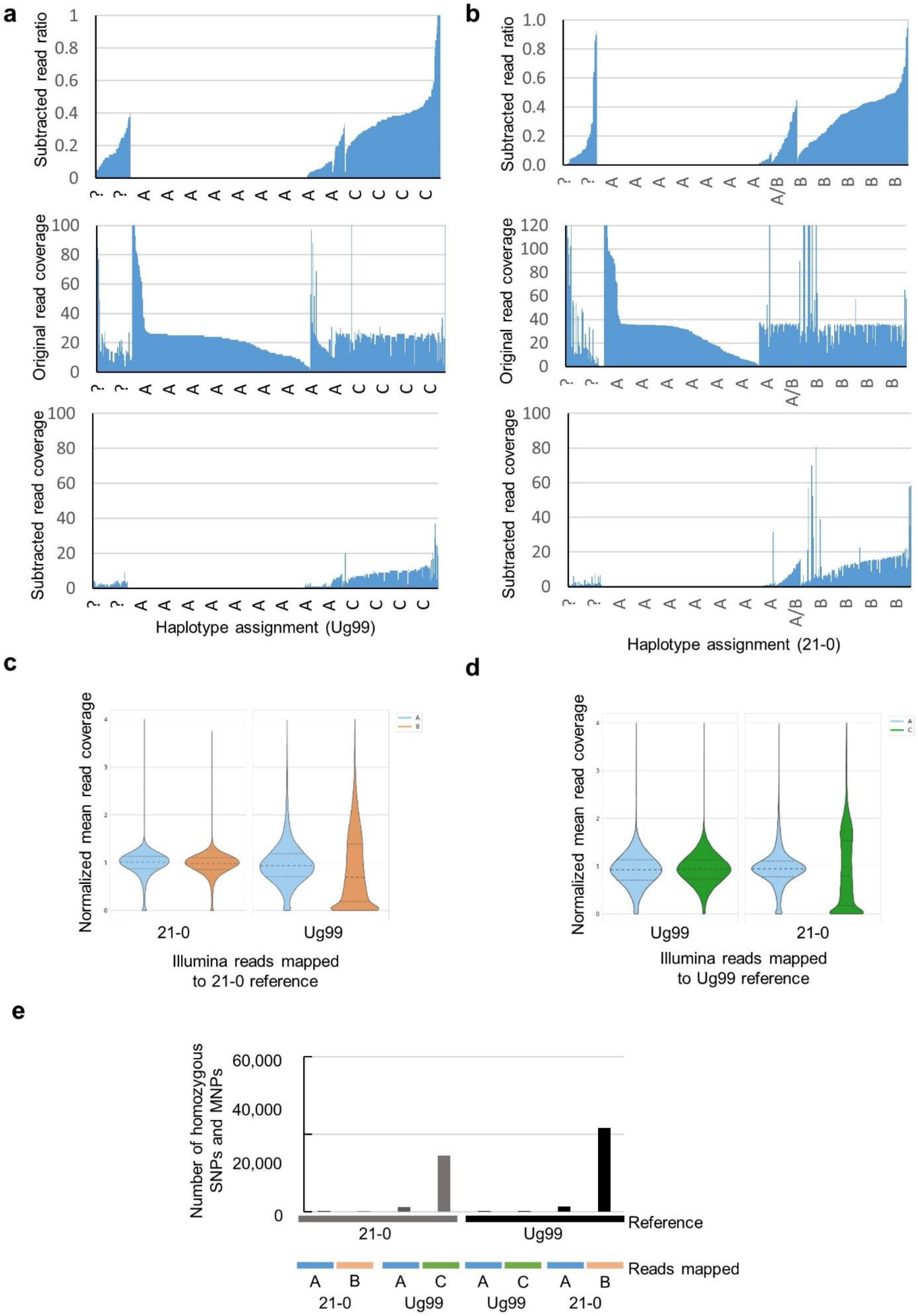

a
Subtracted read ratio
Original read coverage
Subtracted read coverage
Haplotype assignment (Ug99)
b
Haplotype assignment (21-0)
c
Normalized mean read coverage
21-0
Ug99
Illumina reads mapped to 21-0 reference
d
Illumina reads mapped to Ug99 reference
e
Number of homozygous SNPs and MNPs
Reference
Reads mapped

Supplementary Fig. 3 | Haplotype-specific read mapping and SNP calling validates the close identity of haplotype A in *Pgt* Ug99 and *Pgt*21-0. a, Graphs of read depth ratio (top), and read depth of original (middle) and subtracted (bottom) Illumina reads (y-axis) from Ug99 mapped to Ug99 contigs listed by haplotype assignment A, C, A/C chimeras or unassigned (?) (x-axis). **b**, Graphs of read depth ratio (top), and read depth of original (middle) and subtracted (bottom) Illumina reads (y-axis) from *Pgt*21-0 mapped to *Pgt*21-0 contigs listed by haplotype assignment A, B, A/B chimeras or unassigned (?) (x-axis). Data included in Supplementary Data 3. **c,** Violin plots for the distribution of read coverage for haplotype A (blue) and B (orange) after mapping Illumina reads from Ug99 or *Pgt*21-0 to the *Pgt*21-0 assembly. **d,** Violin plots for the distribution of read coverage for haplotype A (blue) and C (green) after mapping Illumina reads from Ug99 or *Pgt*21-0 to the Ug99 assembly. For **c** and **d** y-axis depicts genome coverage calculated in 1 kb sliding windows and normalized to the mean of coverage of each haplotype. Genome coverage shows a normal distribution for self-mapping. Read cross-mapping also shows a normal distribution for haplotype A of Ug99 and *Pgt*21-0 which indicates high sequence similarity. In contrast, a skewed distribution to low genome coverage occurs in the B and C haplotype comparison due to high sequence divergence. **e,** Numbers of homozygous SNPs and MNPs called for various extracted Illumina read sets mapped against the *Pgt*21-0 (grey) or Ug99 (black) reference genome assemblies. Illumina reads were first mapped at high stringency to the corresponding reference genome and then uniquely mapped reads from each haplotype were extracted and used for variant calling. The low number of SNPs detected in the inter isolate comparisons of haplotype A in contrast to the high number of SNPs identified in the B or C haplotype, supports the close identity of A haplotypes in both isolates.

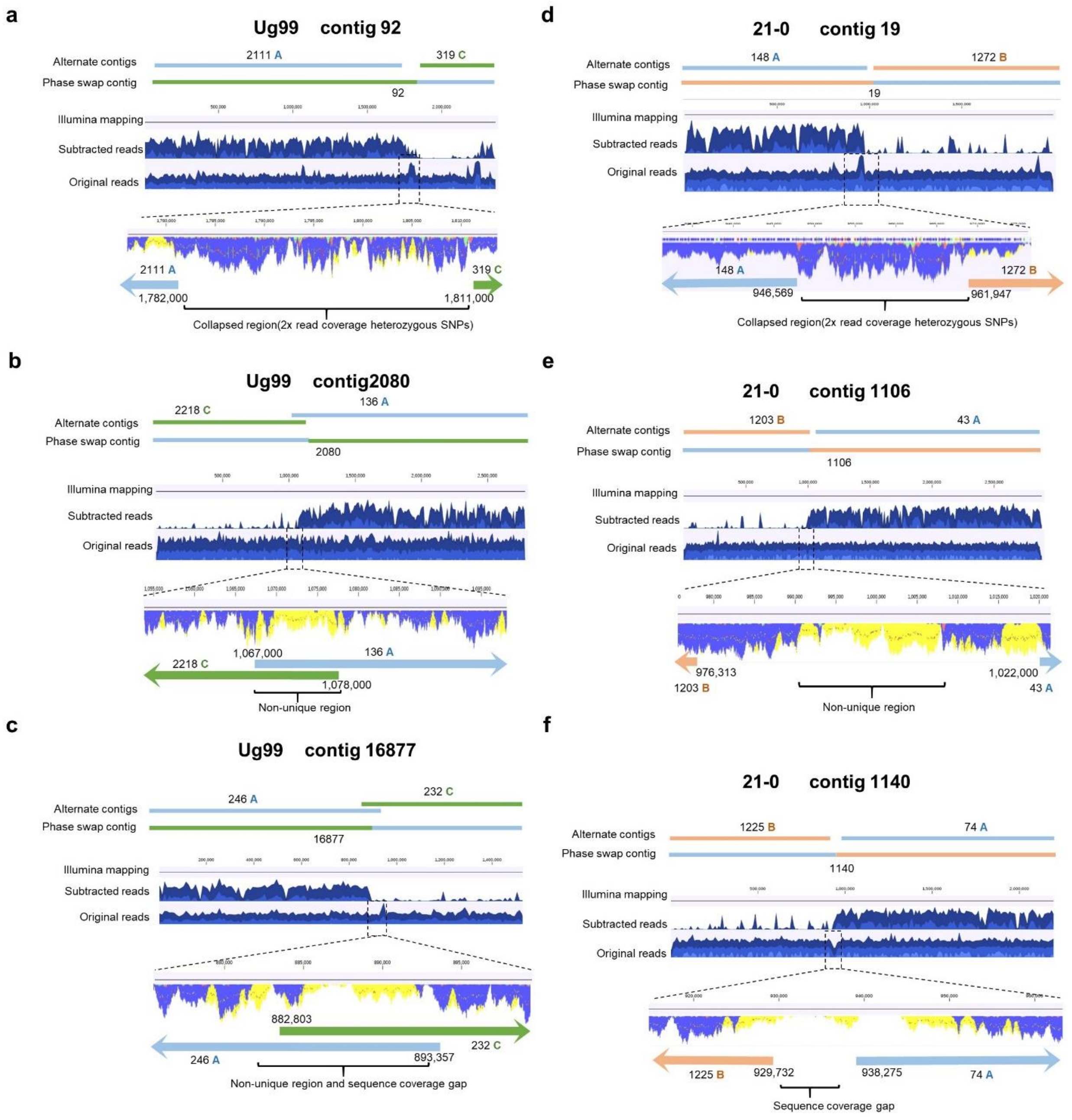

Supplementary Fig. 4 | Examples illustrating the detection and manual curation of phase swap contigs in the *Pgt*21-0 and Ug99 genome assemblies. a to **f,** The top of each figure shows chimeric contigs and alternate contigs colour-coded according to haplotype assignment. The next two tracks show read coverage graphs of subtracted reads and original reads across the phase swap contigs visualized in CLC Genomics Workbench browser (see read subtraction procedure Fig. 3). Zoomed in regions (dotted boxes) show coverage graphs for the phase swap junction regions. Coloured bars indicate SNP frequencies in the underlying reads, and yellow shading indicates non-uniquely mapped reads. Coloured arrows at the bottom shows alignment positions of the alternate contigs to this region with the endpoint coordinates indicated. These examples illustrate scenarios indicative of assembly errors due to collapsed assembly regions showing double overage with heterozygous SNPs (**a, d**), non-uniquely mapped repeats (**b, e**) or coverage gaps after Illumina read mapping (**c, f**).

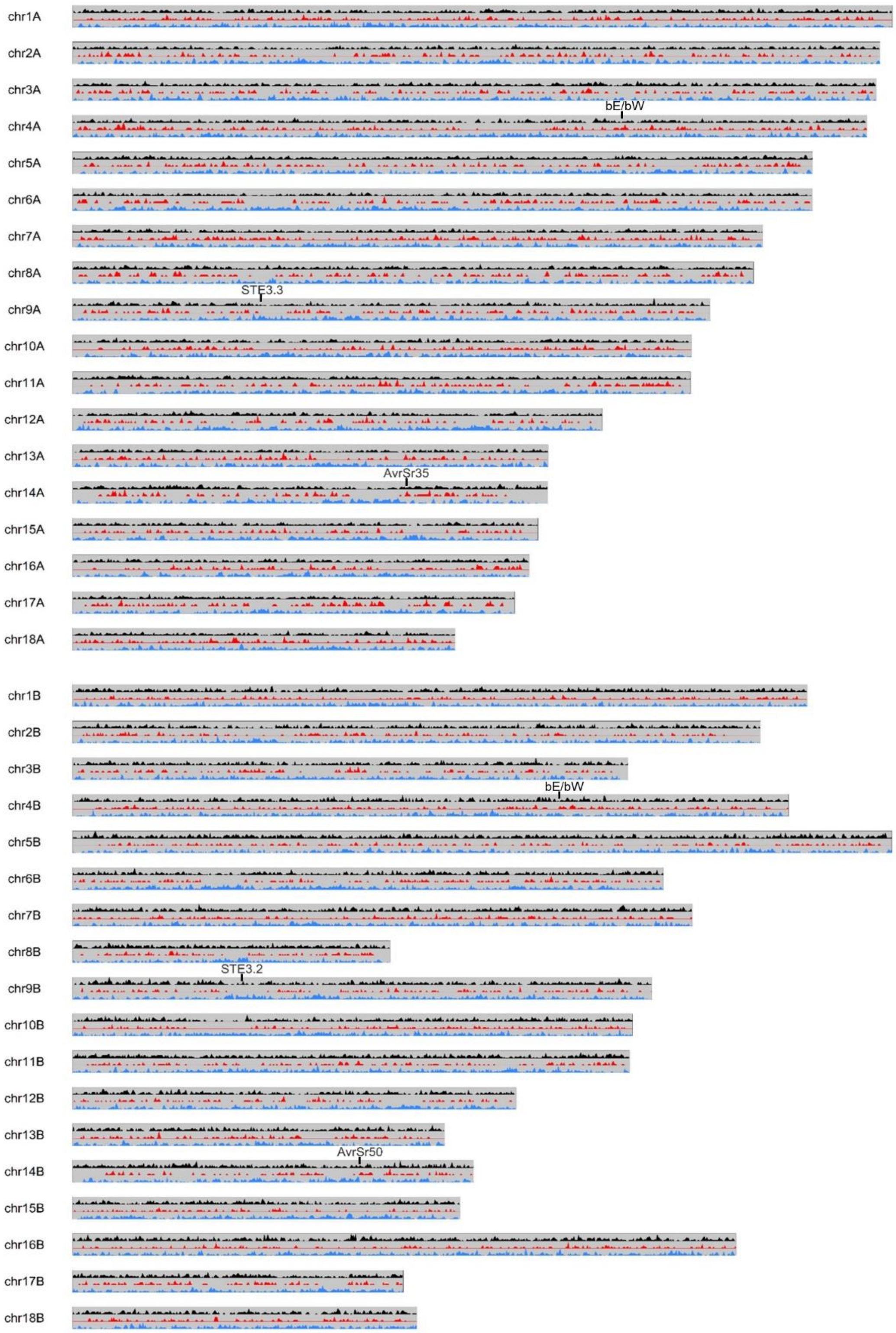

Supplementary Fig. 5 | Gene and repeat density plots for homologous chromosomes in haplotypes A and B of *Pgt*21-0. Top two tracks show density of genes encoding non-secreted (black) or secreted proteins (red) along the chromosomes. Bottom graph shows density of repeat elements (blue). Positions of *bE/bW*, *STE3.2*, *STE 3.3*, *AvrSr50* and *AvrSr35* genes are indicated.

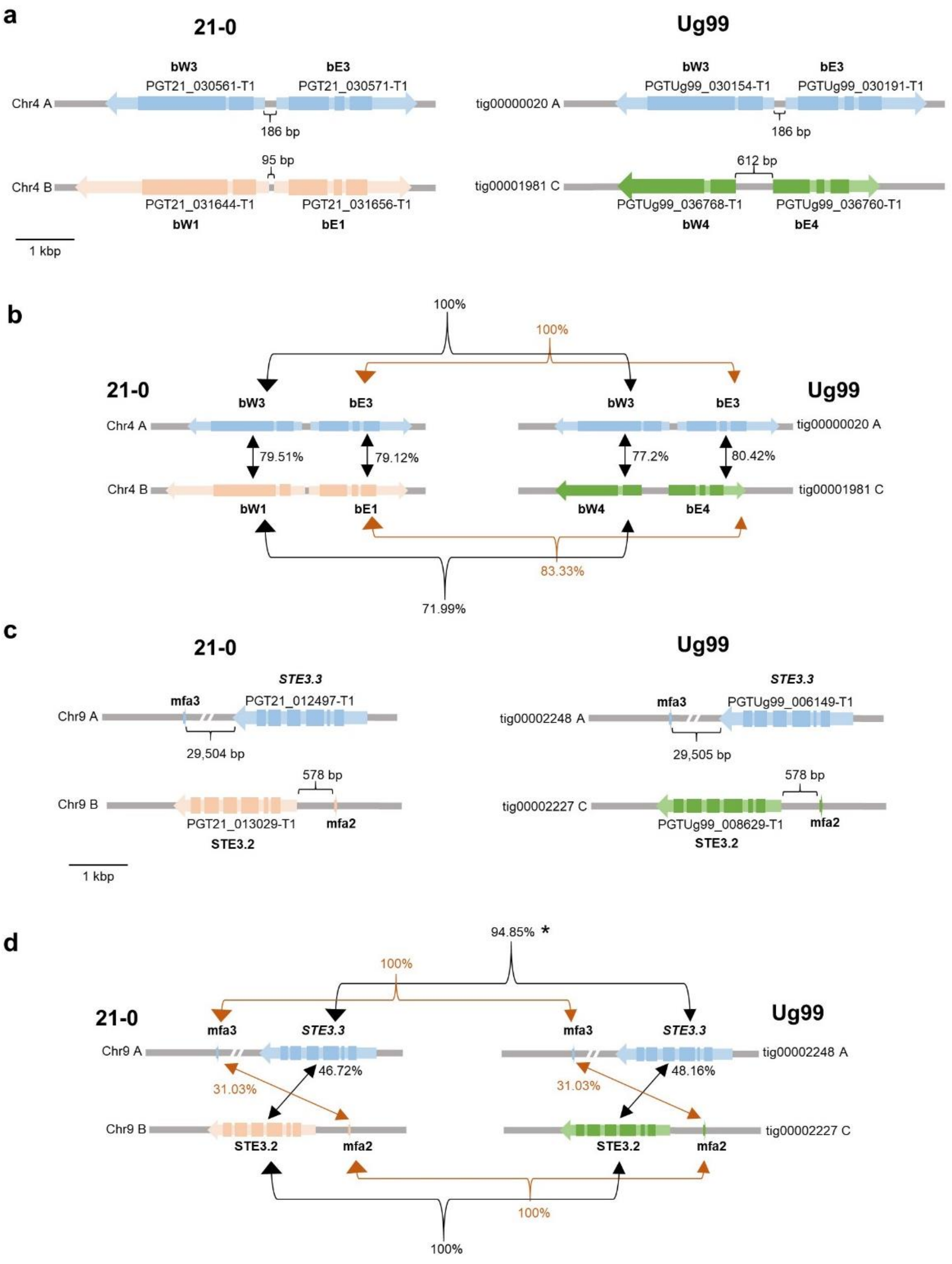

Supplementary Fig. 6 | Structure of mating type loci in *Pgt*21-0 and Ug99. a, The gene transcripts and orientations of the *bE*/*bW* genes from the *b* mating-type locus (chromosome 4) are depicted by light coloured arrows and coding sequences by the darker boxes. Colour coding represents the three haplotypes (A=blue, B=orange, C=green). The distances between predicted gene models are shown. The *bW1*/*bE1* allele is identical to the *bE1*/*bW1* allele previously identified in a North American isolate 75-36-700-3[4]. The *bE2/bW2* allele from 75-36-700-3 was not present in either isolate, which instead contained two additional novel alleles, *bE3*/*bW3* and *bE4*/*bW4*, indicating that this locus is multi-allelic in *Pgt*. **b,** Percentage amino acid identity between predicted proteins encoded by *bE* and *bW* alleles within and between *Pgt* isolates. **c,** Arrangement of the pheromone peptide encoding genes (*mfa2* or *mfa3*) and pheromone mating factor receptors (*STE3.2* and *STE3.3*) at the predicted *a* mating type locus. The two alleles of the *a* locus on chromosome 9 contain either the *STE3.2* (B and C haplotypes) or *STE3.3* (A haplotype) genes from CRL 75-36-700-3[4] in both isolates, consistent with a binary

recognition system. **d,** Percentage amino acid identity between pheromone peptide and receptor alleles within and between *Pgt* isolates. The *STE3.3* allele in Ug99 is identical to that in *Pgt*21-0 except for a 1 bp deletion causing a frameshift and replacement of the last 48 amino acids by an unrelated 24 amino acid sequence resulting in reduced amino acid identity (*).

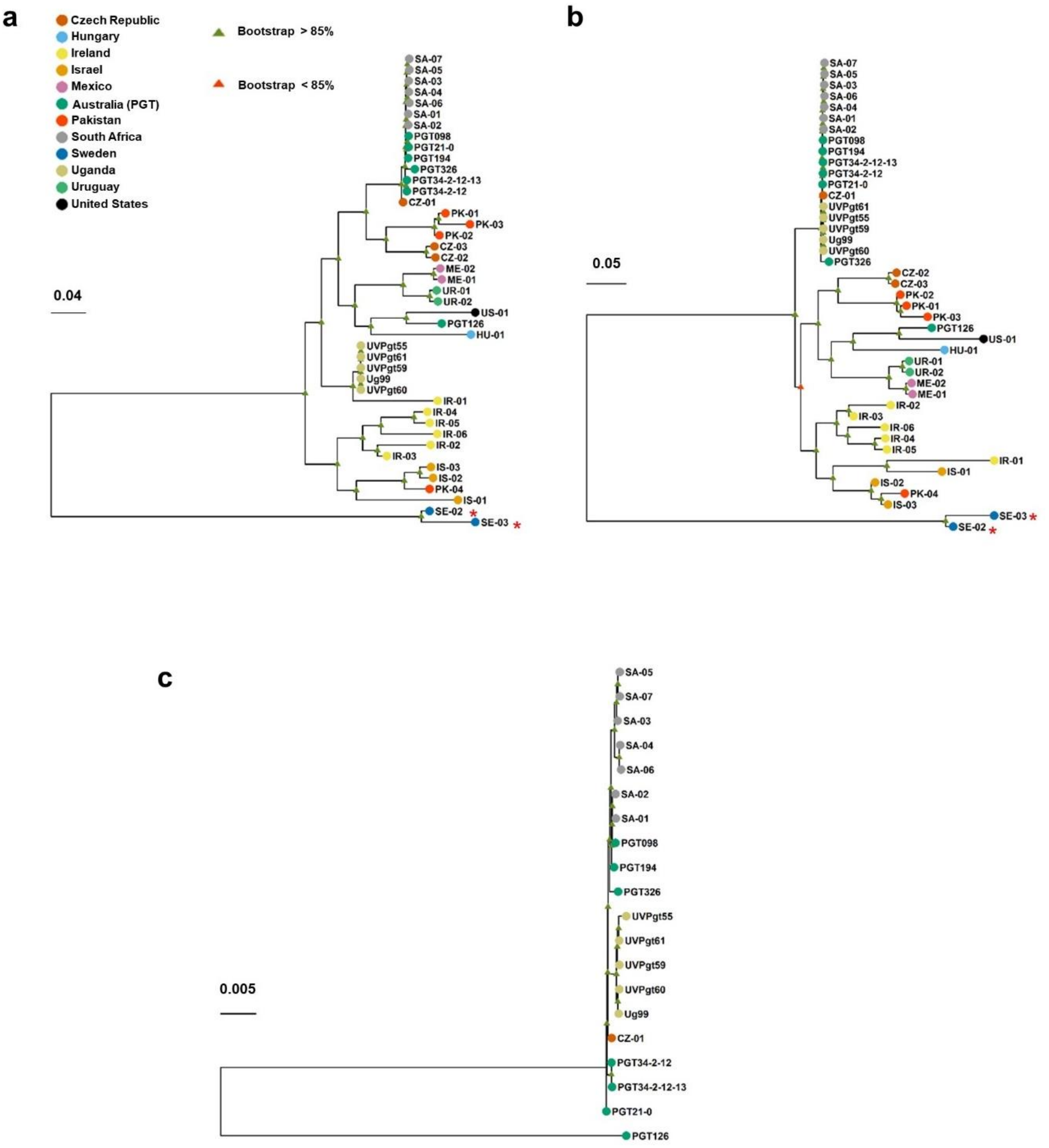

Supplementary Fig. 7 | Phylogenetic analysis of *Pgt* isolates from diverse countries of origin. a, Dendrogram inferred using biallelic SNPs detected against the complete diploid genome assembly of Ug99. **b,** Dendrogram inferred from SNPs detected in haplotype A of Ug99. **c,** Dendrogram inferred using SNPs in haplotype A of *Pgt*21-0 for the South African, Australian and Ug99 lineage isolates that share the A haploid genome, with *Pgt*126 included as an outgroup. Colour key in panel **b** indicates country of origin for all dendrograms. Scale bar indicates number of nucleotide substitutions per site. Red asterisks indicate *P*. *graminis* f. sp. *avenae* isolates.

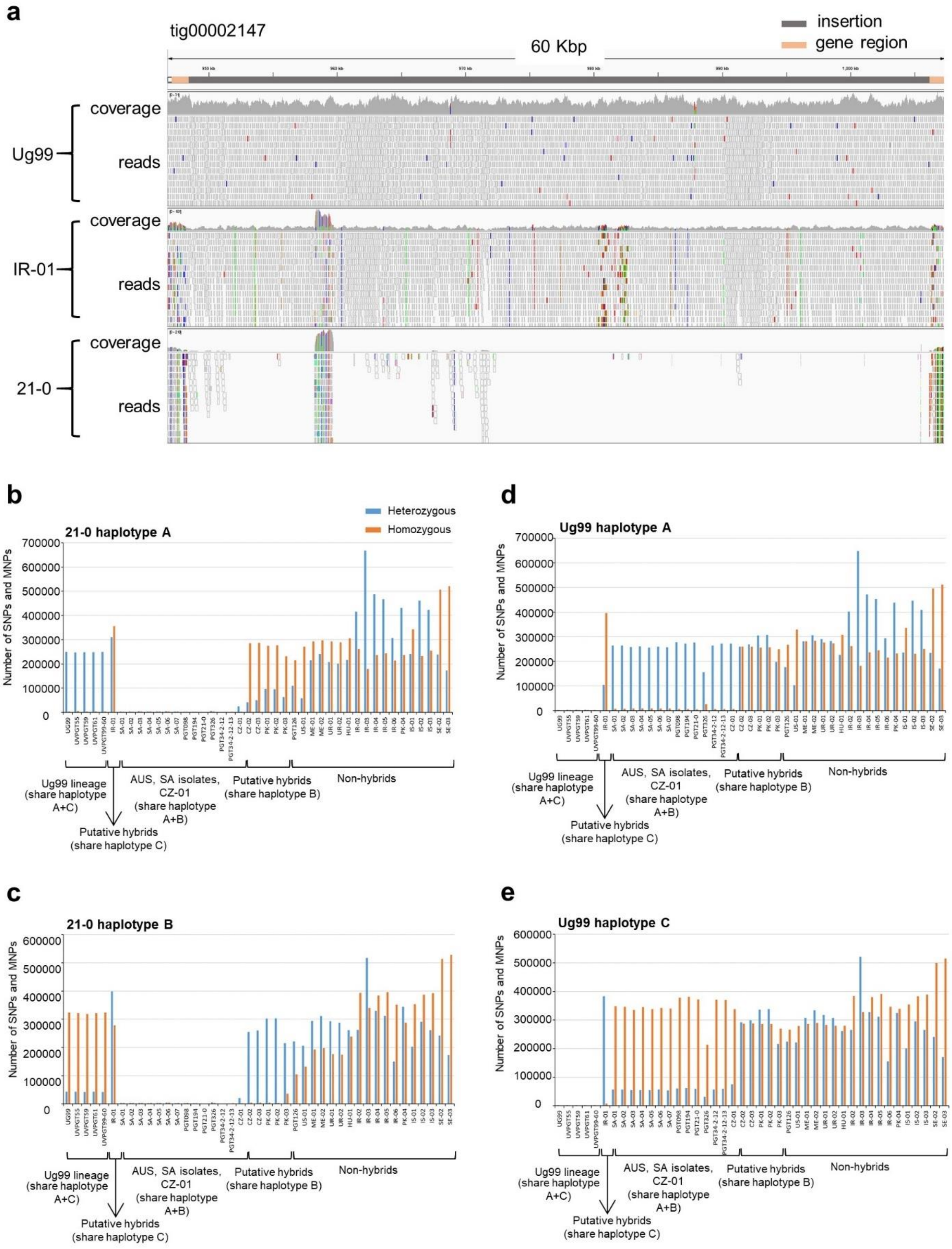

Supplementary Fig. 8 | Putative *Pgt* hybrids that share the B or C haplotypes of *Pgt*21-0 and Ug99, respectively. a, Genome browser view in IGV of a 60 kbp genomic region in haplotype C of Ug99. The top bar shows the *AvrSr35* coding sequences (orange) flanking a 57 kbp-insert (grey). Following tracks illustrate coverage and Illumina read alignments of Ug99, IR-01, and *Pgt*21-0. In contrast to *Pgt*21-0, the genome of IR-01 contains a sequence similar to the 57 kbp insert in Ug99. **b** to **e,** Bar graphs show number of homozygous (orange) and heterozygous (blue) SNPs and MNPs

called against the *Pgt*21-0 or Ug99 A, B and C haplotypes from Illumina read data for 43 *Pgt* isolates used for phylogenetic analysis. Read mapping patterns to each haplotype vary according to the presence or absence of either the A, B or C haplotypes in each isolate. Considering read mapping to the *Pgt*21-0 reference first; for *Pgt*21-0 and the other clonal Australian and South African isolates containing both A and B haplotypes, reads from the A nucleus will map to the A genome and reads from the B nucleus will map to the B genome. A very low number of homozygous SNPs are therefore detected that represent accumulated mutations as this lineage has evolved. For Ug99 and other A+C haplotype isolates in this clonal group, reads from the A nucleus will map to the A genome and again any new mutations appear as a small number of homozygous SNPs in the A genome data set. However, reads from the C genome can map to either the A or B genomes according to sequence similarity. Those reads that map to the B genome will give rise to homozygous SNPs representing divergence between the B and C genomes. However, reads that map to the A genome will give rise to heterozygous SNPs because A genome reads are already mapped to these regions. Thus, for A+C haplotype isolates, we see a small number of homozygous SNPs but a large number of heterozygous SNPs called against the A genome of *Pgt*21-0 (**b**), while the reverse is true for SNPs called against the B genome (**c**). Other isolates that are not hybrids derived from race 21 will have two nuclei that are neither A nor B, and reads from these can map to either the A or B genomes giving rise to high numbers of both heterozygous and homozygous SNPs on each haplotype. Thus, the variation in these patterns of heterozygous and homozygous SNPs on the different haplotypes are indicative of different hybrid relationships. The patterns for CZ-02,03 and PK-01,02 are consistent with them containing haplotype B, with many heterozygous but very few homozygous SNPs called on this haplotype, while IR-01 shows a similar pattern on haplotype C, again indicating that it contains a very similar haplotype.

Supplementary Table 1.

Summary statistics for SMRT sequencing and raw read metrics.

	21-0	Ug99	
	RSII	RSII	Sequel
Number of SMRT cells	17	4	5
Total Bases (Gb)	17.4	2.48	19.66
Number of Reads	1,248,195	317,864	2,634,315
Mean Subread Length (bp)	10,239	7,790	7,591
N50 Subread Length (bp)	16,438	12,080	13,550

Supplementary Table 2.
Summary statistics of Illumina sequencing of *Pgt* isolates in the Ug99 lineage.

Isolate (Pathotype)	150 bp Paired-End Reads	Yield (Mbp)	Mean Quality Score
UVPgt55 (TTKSF)	40,048,658	12,094	31.53
UVPgt59 (TTKSP)	27,204,289	8,216	31.14
UVPgt60 (PTKST)	36,665,018	11,073	31.56
UVPgt61 (TTKSF)*	36,605,359	11,055	31.57
Ug99 (TTKSK)	35,674,381	10,773	31.48

* Virulent on resistance gene *Sr9h*

Supplementary Table 3.
Assembly metrics and quality analysis

Parameters	21-0	Ug99
No. of contigs	410	514
No. of contigs ≥ 50,000 bp	249	333
Total length (Mbp)	176.9	176.0
Total length ≥50000bp (Mbp)	171.8	170.4
Size of Largest contig (Mbp)	5.96	4.40
N50 (Mbp)	1.26	0.97
GC (%)	43.5	43.5
No. of contigs with telomeres	69	26
% of complete BUSCOs	95.8	95.6
% single-copy BUSCOs	8.3	8.9
% duplicated BUSCOs	87.5	86.7
% of fragmented BUSCOs	1.9	1.9
% of missing BUSCOs	2.3	2.5
No. of Bins	44	62
Total length in bins (Mbp)	169	165
No. contigs in bins	225	276

Supplementary Table 4.
Intra and inter-isolate sequence comparison of the *AvrSr50* chromosome haplotypes in Ug99 and *Pgt*21-0.

	Sequence similarity							Structural variation (SV)		
									Total size of variants	
**Isolate comparison**[a]	**Bases aligned (%)**	**Average identity of alignment blocks (%)**	**Overall identity**[b] **(%)**	**Divergence of aligned blocks (%)**	**Total SNPs**	**SNPs/kbp**	**Indels**	**Number of variants >50bp**	**Mbp**	**% of chromosome**
Ug99 A vs *Pgt*21-0 A	99.8	99.93	99.73	0.07	307	0.10	820	29	0.17	2.56
Ug99 A vs Ug99 C	70.82	95.12	67.36	4.88	52,839	25.28	34,563	167	1.3	22.03
21-0 A vs *Pgt*21-0 B	73.12	95.50	69.83	4.50	57,463	22.03	37,070	190	1	14.03
Ug99 C vs *Pgt*21-0 B	78.36	97.27	76.22	2.73	33,655	14.56	21,766	137	1.09	16.74
Ug99 A vs *Pgt*21-0 B	71.87	94.98	68.26	5.02	55,310	26.07	35,314	187	1.25	19.2
21-0 A vs Ug99 C	64.26	95.11	61.12	4.89	54,593	23.82	35,340	150	0.97	14.8

[a] First listed isolate served as reference and second listed isolate served as query for the analysis.

[b] Overall identity is average identity of alignment block multiplied by the proportion of bases aligned.

Supplementary Table 5.

Intra- and inter-isolate sequence comparison of entire haplotypes in Ug99 and *Pgt*21-0.

	Sequence similarity							Structural variation (SV)		
									Total size of variants	
Isolate comparison[a]	Bases aligned (%)	Average identity of alignment blocks (%)	Overall identity[b] (%)	Divergence of aligned blocks(%)	Total SNPs	SNPs/Kbp	Indels	Number of variants >50bp	Mbp	% of genome
21-0 A vs Ug99 A	99.64	99.92	99.56	0.08	9,275	0.10	24,835	491	0.82	0.46
Ug99 A vs Ug99 C	91.52	95.92	87.79	4.08	1,367,911	17.73	851,465	2,571	13.69	7.88
21-0 A vs *Pgt*21-0 B	91.38	97.60	87.55	2.40	1,418,591	17.71	877,814	2,696	15.01	8.56
21-0 B vs Ug99 C	93.44	95.82	91.20	4.18	876,653	11.13	572,042	1,910	11.50	6.69
Ug99 A vs *Pgt*21-0 B	91.52	95.90	87.69	4.10	1,414,244	17.63	877,352	2,648	14.69	8.29
21-0 A vs Ug99 C	91.54	95.81	87.79	4.19	1,371,178	17.77	851,247	2,585	13.88	8.08

[a] First listed isolate served as reference and second listed isolate served as query for the analysis.

[b] Overall identity is average identity of alignment block multiplied by the proportion of bases aligned.

Supplementary Table 6. Chromosomes sizes in *Pgt*21-0

Chromosome	size in A haplotype (bp)	size in B haplotype (bp)
1	6,156,315	6,527,486
2	6,062,178	6,110,382
3	6,034,412	4,933,094
4	5,966,401	6,360,166
5	5,557,100	7,276,977
6	5,553,668	5,248,565
7	5,183,406	5,503,882
8	5,112,795	2,821,965
9	4,787,417	5,140,183
10	4,647,647	4,889,217
11	4,639,132	4,947,173
12	3,976,497	3,939,087
13	3,569,361	3,304,927
14	3,567,101	3,561,970
15	3,495,074	3,444,174
16	3,430,011	5,891,779
17	3,317,526	2,935,361
18	2,873,293	3,063,918
total	83,929,334	85,900,306
	total size A and B	169,829,640

Supplementary Table 7.

Summary of gene annotation

	21-0	Ug99
No. of genes including tRNAs	37,061	37,394
No. of protein coding genes	36,319	36,659
- haplotype A	18,225 (17,786)*	18,593
- haplotype B or C	17,919 (17,718)*	17,621
% of genome covered by genes	34.9	33.6
Mean gene length (bp)	1,667	1,586
No. of secreted protein genes	6,180	6,120
- haplotype A	3,099 (3,071)*	3,212
- haplotype B or C	3,063 (3,046)*	2,857

*No. of predicted genes in contigs assigned to chromosomes

Supplementary Table 8.

Shared and unique gene content between *Pgt* haplotypes

	Haplotypes compared			Haplotypes compared		
	21-0 A	21-0 B	Ug99 C	Ug99 A	21-0 B	Ug99 C
Unique genes	3,369 (18%)	2,668 (15%)	2,950 (17%)	3,529 (19%)	2,774 (15%)	2,950 (17%)
genes shared with one other haplotype	2,492 (14%)	3,165 (18%)	2,543 (14%)	2,976 (16%)	2,664 (15%)	2,827 (15%)
genes shared in three haplotypes	12,364 (68%)	12,086 (67%)	12,128 (69%)	12,088 (65%)	12,481 (70%)	12,082 (69%)
total genes	18,225	17,919	17,621	18,593	17,919	17,621

Supplementary Table 9.

List of primer sequences to amplify flanking and internal regions of the 57 kbp-insert in *AvrSr35*.

Primer ID	Sequence 5'-3'	Amplicon size
MF148 (Forward)	TGCCAAAGTACAAATAGATGACCG	826 bp (with MITE sequence, 1,226 bp)
MF149 (Reverse)	AGATCTTTGAGGTGCTCCCC	
MF150 (Forward)	AGACAGTGTGAAATCAAGTACGT	609 bp
MF151 (Reverse)	CTCATGACAAGGGGCAGGG	
MF152 (Forward)	GCCCTTCAACATTCAGCCTC	678 bp
MF153 (Reverse)	GAGGTGCTCCCCAGGTATTA	

Supplementary References

1. Chen, J., *et al.* Loss of AvrSr50 by somatic exchange in stem rust leads to virulence for Sr50 resistance in wheat. *Science* **358**, 1607-1610 (2017).

2. Salcedo, A., *et al.* Variation in the *AvrSr35* gene determines *Sr35* resistance against wheat stem rust race Ug99. *Science* **358**, 1604-1606 (2017).

3. Duplessis, S., *et al.* Obligate biotrophy features unraveled by the genomic analysis of rust fungi. *Proceedings of the National Academy of Science USA* **108**, 9166-9171 (2011).

4. Cuomo, C.A., *et al.* Comparative analysis highlights variable genome content of wheat rusts and divergence of the mating loci. *G3: Genes, Genomes, Genetics* **7**, 361-376 (2017).
