# Supplementary material for: Emergence of the Ug99 lineage of the wheat stem rust pathogen through somatic hybridisation
Source: Nat Commun. 2019 Nov 7;10:5068. doi: 10.1038/s41467-019-12927-7 (PMC6838127; doi:10.1038/s41467-019-12927-7)
Supplement: Supplementary file 3 — Description of Additional Supplementary Files [file 41467_2019_12927_MOESM3_ESM.pdf]

Description of Additional Supplementary Files

File Name: Supplementary Data 1

Description: Virulence reactions and pathotype assignments of Pgt isolates in the Ug99 lineage. Scores are reported based on the North American wheat differential set

File Name: Supplementary Data 2

Description: Gene synteny output

File Name: Supplementary Data 3

Description: Summary of karyon assignment before breaking chimeric contigs in Ug99 and Pgt21-0

File Name: Supplementary Data 4

Description: List of chimeric contigs and breakpoints

File Name: Supplementary Data 5

Description: Physical linkage of phase swap contigs in the Pgt21-0 assembly to contigs of the same or alternate haplotype within bin or chromosome calculated from Hi-C data

File Name: Supplementary Data 6

Description: Contigs assigned to chromosomes

File Name: Supplementary Data 7

Description: . Metadata for RNAseq libraries of Pgt21-0 used for training gene models in the annotation pipeline

File Name: Supplementary Data 8

Description: Metadata genome coverages after mapping Illumina reads to Pgt21-0 and Ug99 references
